# Massager-Induced Anterior Subcapsular Cataracts and Keratoconus in a Patient With Multiple Epiphyseal Dysplasia

**DOI:** 10.7759/cureus.19095

**Published:** 2021-10-28

**Authors:** Humair Khan, Ahmad Kharsa

**Affiliations:** 1 Department of Ophthalmology and Visual Sciences, University of Texas Medical Branch, Galveston, USA

**Keywords:** multiple epiphyseal dysplasia, extracellular matrix, trauma, keratoconus, massager induced, anterior subcapsular cataracts

## Abstract

Multiple epiphyseal dysplasia (MED) is a phenotypically heterogeneous disease associated with orthopedic abnormalities among other systemic manifestations. While the spectrum of ocular abnormalities in this disorder is yet to be fully reported, MED has been rarely associated in the literature with the development of cataracts and keratoconus. Here, we report a case of bilateral massager-induced anterior subcapsular cataracts and keratoconus in a 46-year-old female with MED. This case presentation aims to prevent similar occurrences of inappropriate massaging device use and highlight potential ocular findings in MED patients.

## Introduction

Massager-induced traumatic cataracts are a rare phenomenon with only two documented cases in the literature [1,2]. Herein, we report the case of a 46-year-old female with a history of multiple epiphyseal dysplasia (MED) and allergic conjunctivitis who developed bilateral anterior subcapsular cataracts (ASCs) and was diagnosed with keratoconus following the use of a supersonic massager device on both eyes. While we believe massager-induced trauma to be the primary etiology underlying our patient's cataracts, we report first such presentation in a MED patient within the English ophthalmic literature.

## Case presentation

A 46-year-old female with multiple epiphyseal dysplasia (MED) and allergic conjunctivitis presented to our clinic for the evaluation of bilateral blurry vision that gradually deteriorated over the past month. Furthermore, the patient reported a significant progressive glare in her vision over the last month. Concurrent with the development of these symptoms, and starting one month prior to presentation, the patient reported using a massage gun over the periocular region to relieve her bilateral ocular discomfort, stress, and frontal/maxillary sinus headaches. The patient applied the device with considerable pressure for a few minutes daily over the past month. However, she stopped using the massaging device after noticing the new onset of blurry vision and glare bilaterally, prompting her presentation to the clinic. Of note, the patient did not use any form of topical ocular steroids and was using antihistamine ocular drops for the management of her allergies. She denied any restricted ocular movements, ocular pain or erythema, flashes, floaters, or other ocular trauma.

**Figure 1:**
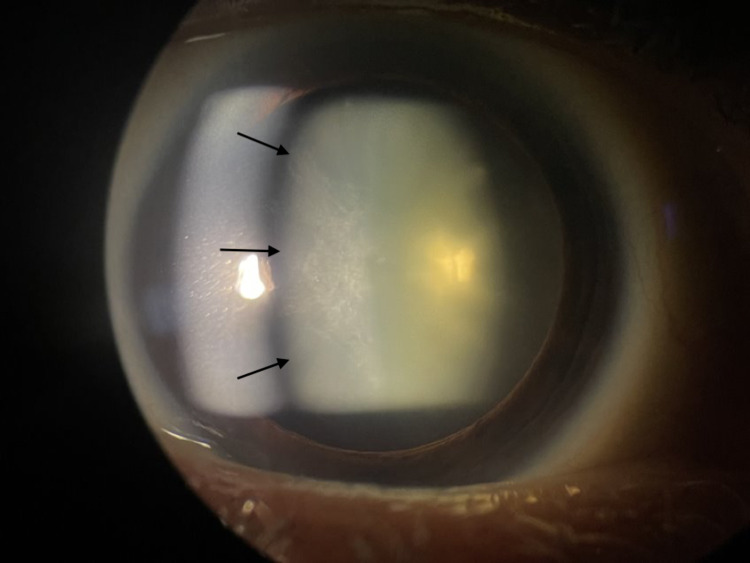
Slit lamp image of the patient’s left eye with a large anterior subcapsular cataract (black arrows).

On examination, the patient’s best corrected visual acuity (BCVA) was 20/40 in the right eye and 20/50 in the left eye. Pupillary response, extraocular motility, and confrontation visual fields were all normal. Intraocular pressure measured 12 mmHg in the right eye and 11 mmHg in the left eye. Slit lamp examination of the anterior segment revealed bilateral anterior subcapsular opacifications consistent with traumatic cataracts (Figure 1). There were no signs of lens dislocation or zonular dehiscence bilaterally. The remainder of the examination for the anterior and posterior segments were within normal limits bilaterally.

**Figure 2:**
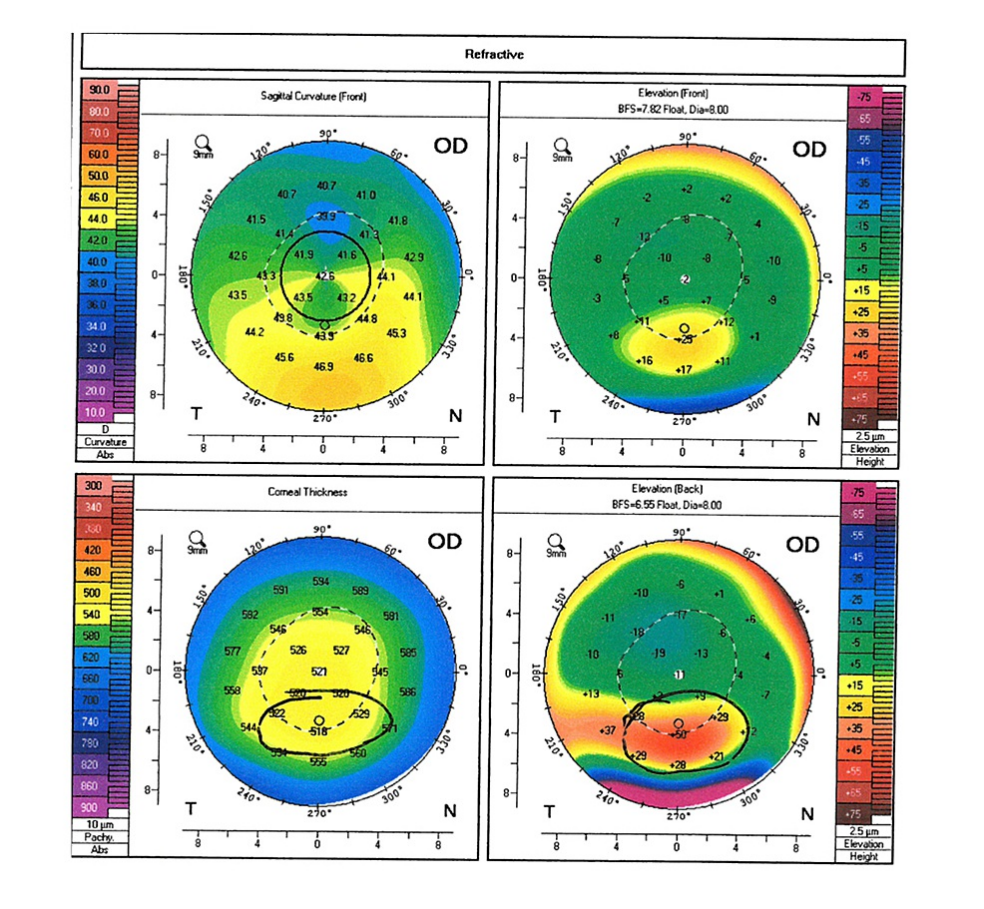
Corneal topography of the patient's right eye. Corneal topography of the right eye revealed a localized region of general inferior steepening bilaterally. Posterior elevation maps showed relative anterior bulging of the posterior cornea consistent with moderate keratoconus.

**Figure 3:**
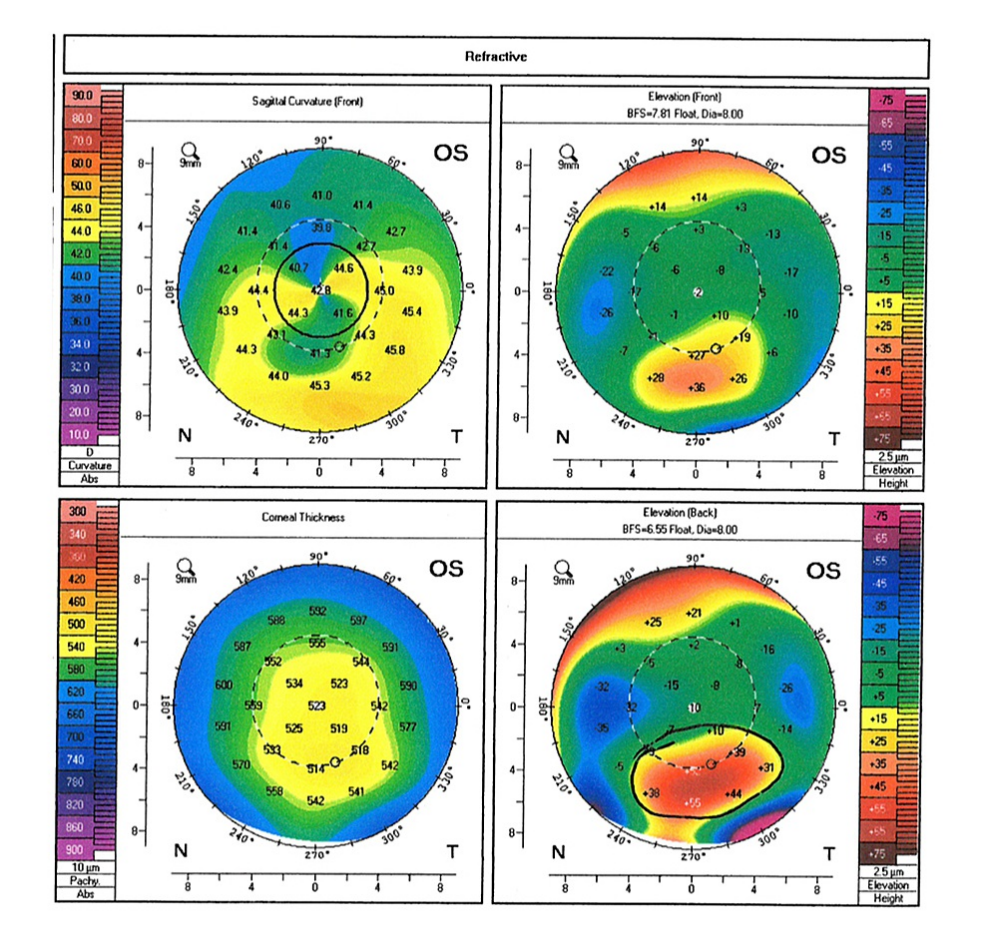
Corneal topography of the patient's left eye. Corneal topography of the left eye revealed a localized region of general inferior steepening bilaterally. Posterior elevation maps showed relative anterior bulging of the posterior cornea consistent with moderate keratoconus.

Given the patient’s history of astigmatism, corneal topography was performed to assess the suitability of toric lens implantation. Surprisingly, while the patient documented no prior ocular history, corneal topography revealed a localized region of generally inferior steepening bilaterally consistent with moderate keratoconus (Figures 2, 3). With these findings, the patient elected to undergo cataract surgery with monofocal intraocular lens implantation. No complications were noted during the surgery and the patient’s best corrected spectacle visual acuity following cataract surgery of the right eye was 20/25. The patient is scheduled for cataract surgery in the left eye in the near future.

## Discussion

MED refers to a spectrum of skeletal disorders that classically affects the epiphyses of long bones, leading to short limbs and early arthritis. The pathophysiology of MED appears to be related to aberrant extracellular matrix protein (EMP), leading to disorganized endochondral ossification and ultimately, destruction of the articular cartilage [3]. While not all of the mutated genes underlying MED have been identified, mutations involving the Cartilage Oligomeric Matrix Protein (COMP) gene account for 70% of the molecularly confirmed MED patients [4]. COMP is a noncollagenous EMP that plays a role in maintaining the structural integrity of cartilage and cross-linking of fibrillar collagens [5]. Although its role in corneal stromal degenerations, such as keratoconus, has not been fully elucidated, mutations in the COMP gene have been associated in the literature with bilateral progressive keratoconus [6]. Furthermore, MED has also been associated with the formation of congenital posterior subcapsular and crenated cataracts [7,8].

We believe that our patient’s history of MED, as a potential predisposing factor to corneal stromal weakness, coupled with her allergic conjunctivitis has driven discomfort in her eyes, leading to the use of the massaging device on her eyes. This massage has facilitated lubrication while providing immediate comfort and relief. However, it has also led to the precipitation of central corneal epitheliopathy and further discomfort, triggering more use of the massaging device. Hence, the development of a vicious cycle occurred. Eventually, the cycle was halted when our patient realized the concomitant worsening of her visual acuity driven by bilateral cataract formation.

Despite other potential etiologies, ocular trauma seems to be the highest risk factor for the development of ASCs [9]. We believe that the continuous and repetitive shockwaves of the massaging device along the plane of the globe have precipitated a contrecoup injury to the lens. Specifically, these distant shockwaves can lead to rapid anterior-posterior shortening accompanied by equatorial stretching, resulting in significant damage to the zonular and lens structures [10].

At the cellular level, and following this traumatic insult, numerous cytokines and growth factor levels, including transforming growth factor β, increase in the aqueous humor and stimulate the lens epithelial cells (LECs) to migrate, proliferate and undergo a process known as epithelial-mesenchymal transition in the damaged areas of the lens. During this process, LECs undergo cytoskeletal rearrangement and transform from epithelial phenotype to mesenchymal phenotype, with deposition of large amounts of extracellular matrix, including collagen and fibronectin. Ultimately, this deposition in situ led to the formation of an opaque subcapsular plaque just beneath the anterior capsule, manifesting as an ASC [10,11].

## Conclusions

In summary, this case showcases an interplay of genetic, environmental, and physiological forces in the pathogenesis of ocular pathology. Nevertheless, further research is needed to elucidate mechanisms underlying keratoconus formation and the impact of skeletal dysplasia on the corneal extracellular matrix. Finally, patient education on the appropriate use of massaging devices is critical to the prevention of similar occurrences of traumatic cataracts.
